# Comparison of Potent Odorants in Traditional and Modern Types of Chinese *Xiaoqu* Liquor (Baijiu) Based on Odor Activity Values and Multivariate Analyses

**DOI:** 10.3390/foods10102392

**Published:** 2021-10-09

**Authors:** Shuang Chen, Jie Tang, Shanshan Fan, Jun Zhang, Shenxi Chen, Yuancai Liu, Qiang Yang, Yan Xu

**Affiliations:** 1 Laboratory of Brewing Microbiology and Applied Enzymology, Key Laboratory of Industrial Biotechnology of Ministry of Education, State Key Laboratory of Food Science and Technology, School of Biotechnology, Jiangnan University, Wuxi 214122, China; shuangchen@jiangnan.edu.cn (S.C.); 6180201007@stu.jiangnan.edu.cn (S.F.); zjchem163@163.com (J.Z.); 2 Key Laboratory of Baijiu Supervision Technology for State Market Regulation, Chengdu 610097, China; 3 Hubei Provincial Key Laboratory for Quality and Safety of Traditional Chinese Medicine Health Food, Jing Brand Research Institute, Jing Brand Co., Ltd., Daye 435100, China; tjjnan@163.com (J.T.); chenshenxi2006@163.com (S.C.); lyc@jingpai.com (Y.L.)

**Keywords:** Chinese *xiaoqu* Baijiu, GC–O, GC–MS, odor activity values (OAVs), multivariate analyses

## Abstract

Predominant odorants in modern and traditional types of Chinese *xiaoqu* liquor (Baijiu) were identified and compared by the combined use of gas chromatography−olfactometry, odor activity values (OAVs), and multivariate analyses. A total of 79 aroma compounds were identified in a typical modern type *xiaoqu* Baijiu (M) and a typical traditional type *xiaoqu* Baijiu (T), 42 of them had OAV > 1 in both M and T samples. The main differences between the two samples were obtained for the concentration of 23 aroma-active compounds. A total of 22 samples made by different brewing processes were analyzed to confirm the differences. Partial least squares discriminant analysis confirmed that 20 compounds could be used as potential markers for discrimination between modern type *xiaoqu* Baijiu and traditional type *xiaoqu* Baijiu. Their difference in content is between 1.5 and 17.9 times for modern type *xiaoqu* Baijiu and traditional type *xiaoqu* Baijiu. The results showed the aroma characteristics of modern and traditional type *xiaoqu* Baijiu clearly and comprehensively, which will provide guidance for modern Baijiu quality control and evaluation.

## 1. Introduction

Traditional fermented foods and beverages (TFFB) have been an important part of the human diet since the beginning of civilization [1]. TFFB remain widely favored by people because of their unique flavor and high nutritional value [2]. TFFB are primarily produced through largely uncontrolled spontaneous inoculation methods [3]. However, many drawbacks in traditional fermentation are directly or indirectly caused by a lack of control. These include low production efficiency, lack of consistency in product quality, and lengthy fermentation times. Thus, modern industrial development is essential in TFFB. Traditional fermentation process evolution and modernization have succeeded in many TFFB, such as soy sauce in the East [4] and cheese in the West [5].

Baijiu, Chinese liquor, is one of the most popular TFFB in China [6], which has thousands of years of history [7]. The annual production of Baijiu reached 7.41 billion liters and the sales revenue of Baijiu reached about 90 billion dollars in 2020, which played an important role in China’s beverage industry [8]. The traditional fermentation and manufacturing methods of Baijiu have existed for centuries and strongly rely on individual operation skills and experience [6]. Traditional type Baijiu is produced using spontaneous fermentation processes in an open environment, and the fermentation temperature varies with the season. With the development of numerous technological innovations, the modern brewing technological process has been applied to Baijiu production, which not only improved the quality of Baijiu but also reduced energy consumption [9,10]. Mechanized operations are used in all stages of the brewing process and information technology is used to control the production environment in a relatively closed and stable environment, which maximally reduces the impact of the environment and operators during the brewing process.

There are very few publications comparing modern type and traditional type Baijiu. Wang et al. [11] separately studied the microbial succession and metabolism changes during Baijiu fermentation of traditional and modern workshops. The study showed that the microbial abundance of the traditional workshop is higher and the environmental microbiota have an important influence on the flavor of Baijiu. Gong et al. [12] explored the differences of volatile and nonvolatile compounds between traditional and mechanical raw Baijiu of sesame-like aroma-type Baijiu. The result showed that except for acids, phenols, and lactones, the total concentrations of the other compounds in traditional raw Baijiu were higher than those in mechanically produced raw Baijiu. Sun et al. [13] characterized 33 and 32 odor-active compounds in traditional and modern type *xiaoqu* Baijiu, respectively, by aroma extract dilution analysis and odor activity values (OAVs). However, the study was focused on only two samples and did not reveal the difference between the aroma compounds of the two types of *xiaoqu* Baijiu. The aroma is the most important characteristic of alcoholic beverages, and the production process is an important factor affecting the aroma profile [14]. So, the characterization of the aroma difference between traditional and modern-type Baijiu is of interest for improving modern brewing technology.

*Xiaoqu* Baijiu, one of the oldest and most popular Baijiu in China, is the first and most successful one to which the fully mechanized modern brewing process has been applied. Therefore, it is necessary to understand fully the similarities and differences of aromatic compounds in the Baijiu manufactured by different production processes. The primary objectives of this study were: (1) to identify the major aroma-active compounds in traditional and modern type *xiaoqu* Baijiu using gas chromatography–olfactometry (GC–O) and gas chromatography–mass spectrometry (GC–MS), (2) to confirm the contribution of those aroma compounds by quantitative analysis and their OAVs, and (3) to verify the aroma difference between the two types of *xiaoqu* Baijiu using multivariate statistical techniques.

## 2. Materials and Methods

### 2.1. Baijiu Samples

A total of 24 *xiaoqu* Baijiu raw liquors were analyzed in this study, including 12 modern type of *xiaoqu* Baijiu and 12 traditional type *xiaoqu* Baijiu. All samples were manufactured by Jing Brand Co., Ltd. (Daye, China) in 2019. The representative modern and traditional type Baijiu samples were chosen by the sensory panel composed of five national Chinese Baijiu tasting judges in Jing Brand Co., Ltd. Among the obtained *xiaoqu* Baijiu samples, one modern type *xiaoqu* Baijiu sample (M) and one traditional type *xiaoqu* Baijiu sample (T) were selected for liquid–liquid extraction (LLE) and GC–O analysis. All the samples were stored at 4 °C until analysis. The brand name is mentioned here for clear labeling, not for advertising purposes. 

### 2.2. Chemicals

Chemical standards and internal standards (ISs) were supplied commercially at a high-purity grade (GC grade, ≥97% purity). Ethyl acetate, isobutyl acetate, isoamyl acetate, pentyl acetate, ethyl propanoate, ethyl 2-methylpropanoate, ethyl butanoate, ethyl 2-methylbutanoate, ethyl 3-methylbutanoate, 3-methylbutyl butanoate, ethyl pentanoate, ethyl hexanoate, propyl hexanoate, hexyl hexanoate, ethyl heptanoate, ethyl octanoate, ethyl nonanoate, ethyl decanoate, ethyl lactate, diethyl succinate, ethyl benzoate, ethyl phenylacetate, 2-phenethyl acetate, ethyl 3-phenylpropanoate, ethyl dodecanoate, ethyl tetradecanoate, 1-propanol, 2-methylpropanol, 1-butanol, 2-butanol, 3-methylbutanol, 1-pentanol, 1-hexanol, 1-heptanol, 2-heptanol, 1-octanol, 3-octanol, 1-octen-3-ol, 1-nonanol, benzyl alcohol, *β*-phenethyl alcohol, 1,1-dimethoxyethane, 2-methylpropanal, 3-methylbutanal, hexanal, decanal, 1,1,3-triethoxypropane, benzaldehyde, benzeneacetaldehyde, 2-pentanone, 2-octanone, acetophenone, 2-pentadecanone, acetic acid, propanoic acid, 2-methylpropanoic acid, butanoic acid, 3-methylbutanoic acid, pentanoic acid, 4-methylpentanoic acid, hexanoic acid, octanoic acid, guaiacol, 4-methylguaiacol, 4-ethylguaiacol, 4-vinylguaiacol, 4-methylphenol, 4-ethylphenol, linalool, *β*-damascenone, geraniol, geranylacetone, *β*-ionone, 2-pentylfuran, furfural, 2-furan methanol, *γ*-nonanolactone, dimethyl trisulfide, 2-thiophenecarboxaldehyde, 2-phenylethyl acetate-D_3_ (IS1), *n*-hexyl-D_13_ alcohol (IS2), 2,2-dimethylpropanoic acid (IS3), 2-octanol (IS4), 2-methoxy-D_3_-phenol (IS5), and lactic acid were purchased from Sigma-Aldrich (Shanghai, China). A C_6_–C_30_ *n*-alkanes mixture (Sigma-Aldrich, Shanghai, China) was used for the determination of retention indices (RIs). Sodium chloride (NaCl), sodium carbonate (Na_2_CO_3_), anhydrous sodium sulfate (Na_2_SO_4_), and hydrochloric acid (HCl) were purchased from China National Pharmaceutical Group Corp. (Shanghai, China). Ethanol (high-performance liquid chromatography (HPLC) grade) was purchased from J&K Scientific Co., Ltd. (Beijing, China). Dichloromethane (CH_2_Cl_2_, HPLC grade, ANPEL Scientific Instrument Co., Ltd., China) was distilled before use. Ultrapure water was obtained from a Milli-Q purification system (Millipore, Bedford, MA, USA).

### 2.3. Extraction of Volatile Compounds

The extraction method was modified from a previous study [15]. The M and T samples (100 mL each) were diluted to 10 vol% with boiled ultrapure water, saturated with NaCl, and then extracted three times using freshly distilled CH_2_Cl_2_ (100 mL each time) in a separatory funnel. The combined extracts were further separated into acidic/water-soluble and neutral/basic fractions with the following method.

The combined extract (about 300 mL) was washed three times with Na_2_CO_3_ (50 mL each time, 0.2 mol/L, pH 10.0) and then washed with 30 mL of saturated NaCl solution. The obtained organic phase, containing the neutral/basic aroma compounds, was named the NBF fraction. The combined aqueous phase was acidified to pH 2.0 with HCl (4.0 mol/L) and extracted three times with freshly distilled CH_2_Cl_2_ (50 mL each time). The combined extract (about 150 mL), containing the remaining acidic aroma compounds, was named the AF fraction. Afterward, each fraction was dried with anhydrous Na_2_SO_4_ overnight and concentrated to a final volume of 0.5 mL under a gentle stream of nitrogen. These concentrated NBF and AF were stored at −20 °C before the GC–O analysis.

### 2.4. Identification of Aroma Compounds Using GC–MS and GC–O

GC–MS and GC–O analyses were performed on an Agilent 6890N GC equipped with an Agilent 5975 mass selective detector and a sniffing port (ODP 2, Gerstel, Württemberg, Germany). The sample was analyzed on both a DB-FFAP column (60 m × 0.25 mm i.d.; 0.25 µm film thickness, Agilent Technologies, Inc., Santa Clara, CA,) and a DB-5 column (30 m × 0.25 mm i.d.; 0.25 µm film thickness, Agilent Technologies, Inc.). High-purity helium (>99.999%) was used as the carrier gas at a constant flow rate of 2 mL/min. The concentrated extract (1 µL) was injected into the GC injector in splitless mode. The GC injector temperature was 250 °C. The oven temperature was held at 50 °C for 2 min, then raised to 230 °C at a rate of 6 °C/min and held for 15 min. The mass spectrometer (MS) was operated in electron ionization mode at 70 eV. The time of solvent delay was 5 min, and the temperature of the ion source was 230 °C. The mass range was set from 35 to 350 amu in full-scan mode.

Four well-trained panelists (two females and two males, 22 years old on average) from the Laboratory of Brewing Microbiology and Applied Enzymology at Jiangnan University were selected for the GC–O study. During a GC run described above, a panelist placed his/her nose close to the sniffing port, responded to the aroma intensity of the stimulus, and recorded the retention time, descriptors, and intensities of the odor peak for each compound. The Osme values reflecting the aromatic intensity of the stimulus based on a six-point scale ranging from 0 to 5 was used for intensity judgment: 0 = none, 1 = very weak, 2 = weak, 3 = moderate, 4 = strong, and 5 = very strong. Every sample was sniffed twice by each panelist, if the aroma was detected six or more times on each column, an odor location was identified. The Osme value for aroma intensity was an average result of the four panelists.

The aroma compounds of the odor location were identified by comparing the aroma, mass spectrum (MS), and RIs to the pure standards (S). RIs were calculated based on the linear retention times of the *n*-alkanes (C_6_–C_30_) in both the DB-FFAP and DB-5 columns under the same chromatographic conditions.

### 2.5. Quantitation of Aroma-Active Compounds by HS-SPME-Arrow Combined with GC–MS

The aroma compounds in all *xiaoqu* Baijiu samples were quantitated using headspace solid-phase microextraction arrow (HS-SPME-Arrow) combined with GC–MS, as described previously [16]. An SPME-Arrow automatic headspace sampling system (CTC Analytics AG, Switzerland) with a 120 μm divinylbenzene/carbon wide range/polydimethylsiloxane (DVB/CAR WR/PDMS) fiber (CTC Analytics AG, Basel, Switzerland) was used for extraction and injection. A total of 5 mL of the diluted *xiaoqu* Baijiu sample (10 vol%) was transferred into a 20 mL headspace glass vial, saturated with 1.5 g NaCl, and spiked with 40 μL mixed ISs (final concentration: IS1, 146 μg/L; IS2, 323 μg/L; IS3, 1 197 μg/L; IS4, 294 μg/L; IS5, 307 μg/L). Tightly capped with a Teflon-faced silicone septum, the sample was equilibrated at 45 °C for 5 min and extracted for 45 min at the same temperature under stirring of 250 rpm. After extraction, the fiber was immediately inserted into the injection port of the GC for desorption at 250 °C for 5 min. The samples were separated on a DB-FFAP column with splitless mode. The oven temperature was held at 40 °C for 2 min, increased at 4 °C/min to 150 °C, then held for 2 min, raised at 6 °C/min to 230 °C and held for 5 min.

Every standard stock solution was prepared by dissolving a standard compound in the model synthetic solution (10% alcohol by volume at pH 4.0) and then diluting to 10 different concentrations. The same amount of ISs as in the *xiaoqu* Baijiu samples was added to the standard solutions, and then the working standard solutions were extracted using HS-SPME-Arrow and detected using GC–MS with the same conditions as for sample analysis. The standard curves were created by plotting the ratio of the peak area of the reference compounds to the corresponding IS against their concentration ratio. The analytical limit of detection (LOD) of aroma compounds was obtained from the lowest concentrations of the analyte standard solutions based on a signal-to-noise ratio of 3. The limits of quantitation (LOQ) was defined as the lowest concentration of the calibration curve based on a signal-to-noise ratio of 10. The accuracy is reported as the percentage recovery of a known amount of target analyte added to the *xiaoqu* Baijiu sample. All analyses were performed in triplicate.

### 2.6. Statistical Analysis

Principal component analysis (PCA) was performed to explore the clustering of the *xiaoqu* Baijiu samples in terms of their aroma compounds. To maximize the separation among samples and to identify the aroma compounds responsible for the separation, partial least squares discriminant analysis (PLS-DA) was used. PCA and PLS-DA were conducted using SIMCA version 14 software (Umetrics, Umearing, Sweden). The heatmap and hierarchical clustering analysis (HCA) were performed using HemI 1.0 (The CUCKOO Workgroup, http://hemi.biocuckoo.org, accessed on 4 September 2021).

## 3. Results and Discussion

### 3.1. Identification of Aroma Compounds in Xiaoqu Baijiu Using GC–O

By extraction with CH_2_Cl_2_, an organic phase was obtained that exactly showed the typical *xiaoqu* Baijiu aroma when sniffed on a filter paper by the panelists. Thus, it was appropriate and reliable to be used to analyze the profile of aroma compounds in *xiaoqu* Baijiu. To facilitate GC–O analysis and compound identification, the extract obtained was further separated into AF and NBF fractions to reduce complexity as previously described [15]. To identify the constituents responsible for the odors, aroma areas were first located using GC–O. The RI of each odorant regions on the DB-FFAP and DB-5 columns was calculated. Then, the aroma quality and odor descriptor of each odorant were determined by comparison to a Chinese Baijiu flavor database (an in-house database consisting of more than 900 odor-active standard compounds). The identities of the tentatively assigned aroma compounds were finally confirmed by comparing their MS data (EI mode) with those of the standard reference compounds.

**Figure 1:**
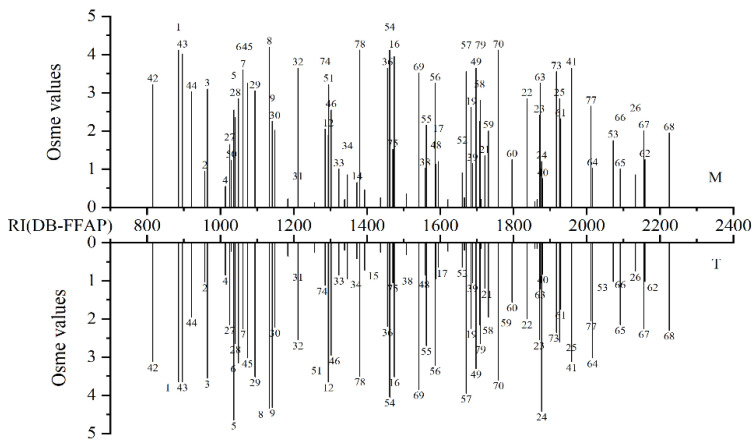
Osme values of aroma extract from M and T samples by GC-O and GC-MS analyses (Annotation: Number on peak corresponds to Table 2, the abscissa represents the retention index of the compounds on the DB-FFAP chromatographic column, and the ordinate represents the aroma intensity of the compounds.).

As shown in Table 1, a total of 79 odorants were located in the M and T samples in this study. The results showed that both liquors had a similar aroma profile, but the Osme values varied (Figure 1). Most of these components are well-known odorants in Baijiu [7,17,18], but 36 of them have not been reported in a previous study of *xiaoqu* Baijiu [13]. Among the 79 aroma compounds, 26 esters, 15 alcohols, 12 aldehydes and ketones, nine acids, six phenols, five terpenoids, four furans, and two sulfur compounds were detected in the M and T samples.

The dominant group of odorants identified was esters, particularly ethyl esters. The most important esters identified were ethyl acetate, isoamyl acetate, ethyl 2-methylpropanoate, and ethyl octanoate with Osme values above 3.00 in both samples, contributing a strong fruity note. In general, the T sample had higher Osme values for most esters than the M sample, such as ethyl butanoate (Osme values = 2.55; 4.65), ethyl pentanoate (Osme values = 2.25; 4.32), ethyl hexanoate (Osme values = 1.90; 3.65), and ethyl 3-phenylpropanoate (Osme values = 1.20; 4.42). However, some other esters had higher Osme values in the M sample. They were ethyl acetate (Osme values = 4.12; 3.65), ethyl 3-methylbutanoate (Osme values = 3.60; 2.25), ethyl octanoate (Osme values = 3.95; 3.52), ethyl decanoate (Osme values = 2.62; 2.25), and ethyl phenylacetate (Osme values = 2.85; 2.00).

The second dominant group of aroma compounds identified were alcohols. Among the alcoholic compounds identified in the M sample, 3-methylbutanol, 1-octen-3-ol, and *β*-phenethyl alcohol showed the highest Osme values of 3.65, contributing whiskey, mushroom, and honey odors, respectively. However, 2-methylpropanol with the Osme value of 3.52 was the most important aroma contributor in the T sample.

Based on GC–O and GC–MS, eight aroma aldehydes were detected. Aromatic aldehydes seemed to be important in *xiaoqu* Baijiu, where 2-methylpropanal (Osme values = 3.22; 3.12), 1,1-dimethoxyethane (Osme values = 4.02; 3.65), hexanal (Osme values = 3.25; 3.02), and benzeneacetaldehyde (Osme values = 3.65; 3.30) had high Osme values above 3.00 and contributed to malty, fruity, green, and honey odors, respectively. In this study, four ketones were identified. Among them, 2-octanone with soap aroma showed the highest Osme values of 3.22 and 3.20 in M and T, respectively. All aldehydes and ketones had higher Osme values in the M sample than in the T sample except 1,1,3-triethoxypropane.

The important acids in M and T with high Osme values were acetic acid (Osme values = 4.12; 4.05), 2-methylpropanoic acid (Osme values = 3.25; 3.22), butanoic acid (Osme values = 3.55; 3.95), 3-methylbutanoic acid (Osme values = 2.25; 2.15), pentanoic acid (Osme values = 2.00; 1.95), and hexanoic acid (Osme values = 2.32; 1.75). Acids are important components in the quality and taste of alcoholic beverages. Most of the acids in Baijiu are produced by yeast during fermentation, followed by oxidation of alcohol and aldehyde [19]. Acetic acid contributed vinegar aroma while 2-methylpropanoic acid, butanoic acid, 3-methylbutanoic acid, pentanoic acid, and hexanoic acid gave a sweaty odor. Phenols could be important contributors to Baijiu flavor due to their low odor threshold. Among the six phenolic compounds, the Osme values of guaiacol (Osme values = 3.25; 1.22) and 4-ethylguaiacol (Osme values = 0.95; 2.15) varied significantly between the M and T samples. Five terpenoids were detected in GC–O. Terpenoids not only have a positive aroma contribution to Baijiu flavor but also have healthy physiological activities [17,20]. Linalool (Osme values = 3.52; 3.85), *β*-damascenone (Osme values = 4.12; 3.60), and *β*-ionone (Osme values = 3.55; 2.35) could be important to the aroma of *xiaoqu* Baijiu because of the high Osme values. Sulfur compounds are very important for Baijiu because of their low thresholds. They are mainly produced by the degradation of sulfur-containing amino acids [21]. Two sulfur compounds, dimethyl trisulfide (Osme values = 4.12; 3.50) and 2-thiophenecarboxaldehyde (Osme values = 2.80; 2.65), were identified in both samples.

### 3.2. Quantitation and OAVs of Aroma-Active Compounds in Xiaoqu Baijiu

**Figure 2:**
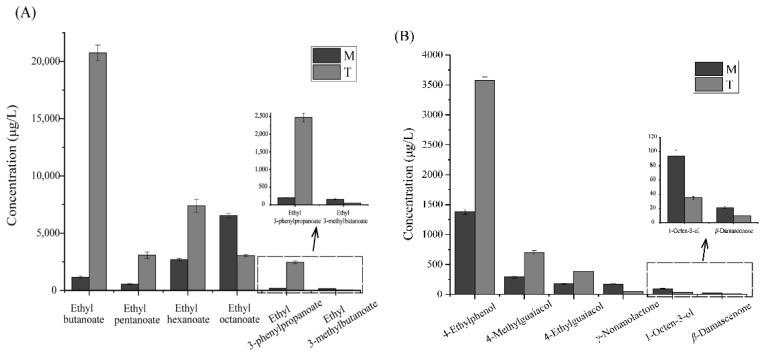
Concentrations of some different aroma-active compounds in M and T samples. In the Figure 2, (**A**) is the concentration difference between ester compounds, and (**B**) is the concentration difference between others.

To gain a deeper insight into the aroma-active compounds of *xiaoqu* Baijiu, the 79 odorants detected using GC–O were quantitated by constructing standard curves for the M and T samples. The quantitative ion, IS, calibration curve, recovery, and LOQ of each aroma compound are shown in Table 2 and Figure 2. Quantitative results showed that 1,1-dimethoxyethane, ethyl acetate, 1-propanol, 2-butanol, 2-methylpropanol, 3-methylbutanol, and acetic acid showed relatively high concentrations (above 100 mg/L) in both samples.

It is known that the contribution to Baijiu depends not only on the concentration of an odorant but also its odor threshold. So, for further confirmation of the contribution of aroma-active compounds, their OAVs were calculated. The OAV was obtained from a compound concentration divided by its odor threshold [22]. Herein, the majority of the threshold values were taken from the literature [23,24,25]. As shown in Table 3, 42 compounds yielded an OAV ≥ 1 in both M and T samples, which indicated that these odorants may have a major contribution to the characteristic aroma. The most important aroma compounds (OAV ≥ 100) in M and/or T were ethyl octanoate (OAVs = 507.20; 235.88), 3-methylbutanal (OAVs = 307.74; 190.36), *β*-damascenone (OAVs = 178.98; 78.11), dimethyl trisulfide (OAVs = 161.22; 116.14), 1,1-dimethoxyethane (OAVs = 140.03; 90.33), isoamyl acetate (OAVs = 96.88; 132.29), ethyl hexanoate (OAVs = 48.52; 133.74), ethyl pentanoate (OAVs = 20.35; 114.76), and ethyl butanoate (OAVs = 14.21; 254.49). These results revealed the important aroma contribution of esters, especially ethyl esters, for *xiaoqu* Baijiu liquors. Esters, which contribute a strong fruity odor, were also one of the most important aroma groups for other alcoholic beverages, such as wine [26], rice wine [27], and other types of Baijiu [7,15,25,28]. Besides, 3-methylbutanal and acetal ranked at the second and fifth places, respectively, according to their OAV. It is worth noting that the content of *β*-damascenone and dimethyl trisulfide was not high but the OAVs ranked at second and third places among all aroma compounds in *xiaoqu* Baijiu. This is because their thresholds are present at trace amounts in Baijiu [23,25]. *β*-Damascenone giving a honey note was previously reported as a key odorant with high OAV in the light aroma type of Baijiu [25,29]. Dimethyl trisulfide, exhibiting cabbage notes, was one of the key aroma compounds in Zhima aroma-type Baijiu and Moutai aroma-type Baijiu [24,30]. The other important odor-active volatiles with OAVs greater than or equal to 10 in M and/or T were ethyl acetate (OAVs = 36.49; 33.53), ethyl 3-methylbutanoate (OAVs = 22.48; 6.56), hexanal (OAVs = 18.21; 13.76), pentanoic acid (OAVs = 17.30; 19.85), 1-octen-3-ol (OAVs = 15.37; 5.70), 1-propanol (OAVs = 12.53; 15.89), linalool (OAVs = 12.39; 14.57), and 2-methylpropanol (OAVs = 8.35; 11.57). Compounds, including ethyl 2-methylpropanoate, ethyl 2-methylbutanoate, guaiacol, 2-butanol, 1-butanol, 3-methylbutanol, ethyl decanoate, ethyl dodecanoate, acetic acid, 2-methylpropanoic acid, butanoic acid, 3-methylbutanoic acid, 4-methylpentanoic acid, hexanoic acid, *β*-ionone, ethyl lactate, 4-vinylguaiacol, 4-ethylphenol, and 2-phenethyl acetate, were other aroma contributors.

### 3.3. Confirmation of the Key Compounds Related to the Aroma Profile Differences between Modern and Traditional Type Xiaoqu Baijiu

As concluded above, the odorant compositions of M and T infusions were similar, but the Osme value and OAV of each substance were different. However, these were just the results based on two samples, not enough to represent all the aroma characteristics of modern type *xiaoqu* Baijiu and traditional type *xiaoqu* Baijiu. To determine if the differences between M and T samples can also be found in an additional 22 different types of *xiaoqu* Baijiu samples, which were randomly selected from the modern and traditional workshops, the 79 aroma compounds were quantitated. Then, PCA and PLS-DA analyses were conducted based on the concentration of the aroma compounds in the 22 *xiaoqu* Baijiu samples.

**Figure 3:**
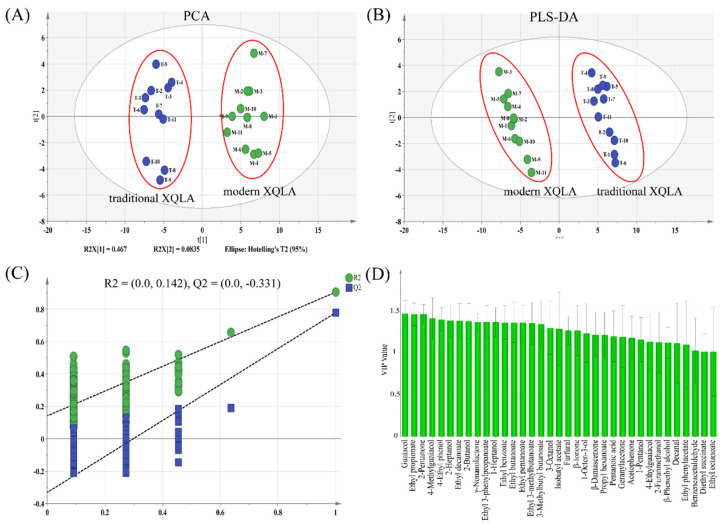
(**A**) PCA (Principal component analysis) scores scatter plot for modern xiaoqu Baijiu and traditional xiaoqu Baijiu samples (PC1 46.7%, PC2 8.35%). (**B**) PLS-DA (partial least squares discriminant analysis) scores scatter plot for different samples (R2Y = 0.993 and Q2 = 0.978). (**C**) The validate model of the PLS-DA model. (**D**) The variable importance for projection (VIP) plot (VIP > 1).

PCA was performed to show a trend of intergroup separation on the scores plot of data obtained in both positive and negative modes. The PCA results showed that five principal components (PCs) with eigenvalues higher than one were obtained from the data sets, whose cumulative variance proportion was 74.5%. PC1 accounted for 46.7% of the total variance while PC2 explained 7.02%, which explained 55.1% of the total variance in the data set. As shown in Figure 3A, it was found that 22 *xiaoqu* Baijiu samples were well separated. PC1 clearly distinguished modern type *xiaoqu* Baijiu and traditional type *xiaoqu* Baijiu, traditional type *xiaoqu* Baijiu was on the positive side of PC1, while modern type *xiaoqu* Baijiu appeared on the negative side, indicating that there were obvious differences in aroma compounds’ features.

To maximize separation and identification of the aroma compound markers between the two types of *xiaoqu* Baijiu, a PLS-DA model was created for the 22 *xiaoqu* Baijiu samples based on the concentration data set. This type of supervised pattern recognition method provides a deeper analysis of the main matrix characteristics of samples and differs from PCA by the addition of grouping variables that indicate the category of samples. As shown in Figure 3B, the two types of *xiaoqu* Baijiu samples were well distinguished by a dependable PLS-DA model. The explained variation (R^2^Y) and the predictive capability (Q^2^Y) for the model were 0.993 and 0.978, respectively, which indicated that the model was excellent because Q^2^Y was close to 1. In addition, permutation tests (*n* = 200) were performed to evaluate whether the discriminant models were overfitting the data [32]. The permutation tests randomly rearranged the experiments by changing the sort order of the classification variables (Y) and randomly assigned Q^2^Y up to 200 times. The low intercepts (R^2^ = 0.142, Q^2^ = −0.331) were an indication of the validity of the original model. The R^2^ and Q^2^ values of the permutation test revealed that the initial model outperformed the randomly permuted models (Figure 3C). Variables with absolute variable importance for projection (VIP) values greater than 1 were selected as potential markers explaining the differences. As shown in Figure 3D, a total of 35 compounds had a VIP value greater than 1, and 20 out of the 35 compounds were important contributors to the aroma profile of *xiaoqu* Baijiu, which indicated that they were highly relevant to the difference between modern type *xiaoqu* Baijiu and traditional type *xiaoqu* Baijiu samples. These 20 substances cover most of the different substances obtained by comparing the concentrations of the M and T samples. This showed that the multivariate statistical analyses of 22 samples verified the results of Section 3.2.

**Figure 4:**
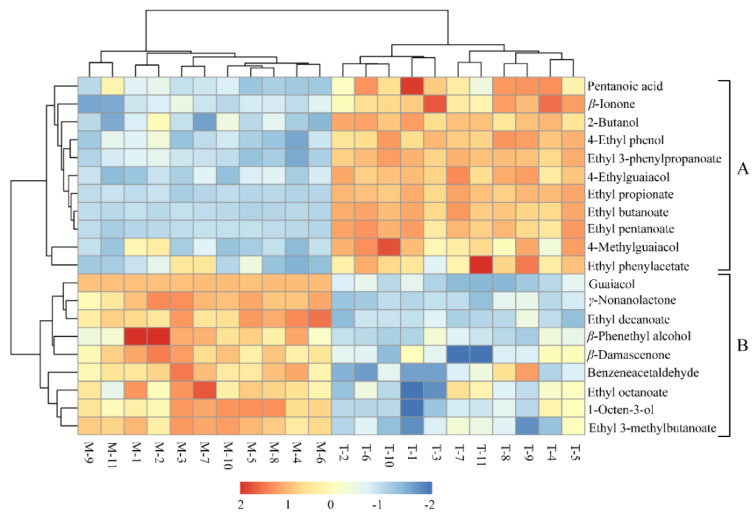
Heat map of the key aroma compounds maker between modern type *xiaoqu* Baijiu and traditional type *xiaoqu* Baijiu.

To compare further the content of each substance in the two types of *xiaoqu* Baijiu, the concentrations of 20 potential aroma-active compounds in the samples from both the modern type *xiaoqu* Baijiu and traditional type *xiaoqu* Baijiu were used to prepare HCA analysis, and the color (from light to dark) indicated the concentration change from low to high. As shown in Figure 4, the HCA clearly classified the aroma markers into two clusters. Cluster A consists of the aroma-active compounds with significantly higher concentrations in traditional type *xiaoqu* Baijiu than in modern type *xiaoqu* Baijiu, which included five ethyl esters (ethyl 3-phenylpropanoate, ethyl propanoate, ethyl butanoate, ethyl pentanoate, and ethyl phenylacetate), three phenols (4-ethylphenol, 4-ethylguaiacol, and 4-methylguaiacol), pentanoic acid, *β*-ionone, and 2-butanol.

The ethyl esters in Baijiu were mainly produced in the fermentation or aging stages through the esterification of acids and alcohols [7,33]. This study found that the contents of propanoic acid, butanoic acid, and pentanoic acid in traditional type *xiaoqu* Baijiu were higher than those in modern type *xiaoqu* Baijiu, so the organic acids had a significant influence on the yield of aroma esters. Phenols, as an important trace component in Baijiu, play an important role in the aroma, taste, and stability of Baijiu [34]. It was reported that phenols come from the thermal degradation of lignin in brewing raw materials or are produced by amino acids and ferulic acid under the action of microorganisms [35,36]. The brewing materials of modern type *xiaoqu* Baijiu and traditional type *xiaoqu* Baijiu are the same, therefore, it may be that the different types and abundance of microorganisms caused the higher content of phenols in traditional type *xiaoqu* Baijiu. *β*-Ionone with a floral aroma is also a very important aroma compound and bioactive substance in Baijiu [37]. Besides, 2-butanol is one of the higher alcohols in Baijiu, it is mostly synthesized by microorganisms (mainly *Saccharomyces cerevisiae* and bacteria) during fermentation via catabolic and anabolic pathways [38,39]. The fermentation temperature of traditional type *xiaoqu* Baijiu is highly susceptible to climatic conditions, but the fermentation temperature of modern type *xiaoqu* Baijiu is controlled at a suitable temperature, which could effectively inhibit the production of higher alcohols [13]. Cluster B consists of the aroma-active compounds with significantly higher concentrations in modern type *xiaoqu* Baijiu than in traditional type *xiaoqu* Baijiu, which included three esters (ethyl 3-methylbutanoate, ethyl octanoate, and ethyl decanoate), guaiacol, *γ*-nonanolactone, *β*-phenethyl alcohol, *β*-damascenone, benzeneacetaldehyde, and 1-octen-3-ol. Not surprisingly, the acids content corresponding to these three esters in modern type *xiaoqu* Baijiu was also higher than in traditional type *xiaoqu* Baijiu. *β*-Phenethyl alcohol with a rose-like aroma is a very important aroma compound in Baijiu. The processes by which the key odorant is formed have been well-established and found to be influenced by various yeast species present during the fermentation of liquor from cereal and legume materials [40]. *β*-Damascenone, presenting honey and floral aromas, was previously reported as an important odorant in whiskey, rum, brandy, and Baijiu [25,28,41,42,43]. Luigi and Peter [43] assumed that cereals could contain the precursor (3-hydroxy-7,8-dihydro-*β*-ionol) leading to the generation of *β*-damascenone during fermentation and/or distillation.

## 4. Conclusions

In summary, a total of 79 aroma compounds were identified using GC–O and GC–MS in both M and T samples. Among them, 42 aroma compounds were further recognized as the important aroma-active compounds in both M and T owing to their relatively high OAVs. Twenty-three aroma-active compounds might be the potential compounds responsible for the differences in the aroma profile between M and T samples because of a concentration ratio higher than 1.5. Moreover, to determine if the 23 aroma-active compounds can represent the difference in aroma compounds’ profile between traditional type *xiaoqu* Baijiu and modern type *xiaoqu* Baijiu, multivariate analyses of the 22 *xiaoqu* Baijiu samples made by different brewing processes were applied, including PCA, PLS-DA, and HCA. Twenty odor-active compounds were confirmed as potential flavor markers for the differentiation of modern type *xiaoqu* Baijiu and traditional type *xiaoqu* Baijiu. The results in this study will help improve the flavor quality and processing technology of *xiaoqu* Baijiu.

## Figures and Tables

**Table 1:** Aroma compounds in Chinese *xiaoqu* Baijiu identified by gas chromatography-olfactometry (GC-O) and gas chromatography-mass spectrometry (GC-MS).

No.	Compound	RI ^a^		Odor ^b^	Fraction ^c^	Identification	Osme Values	
			DB-FFAP			DB-5	Mean ^d^	M	T
	**Esters**							
1	Ethyl acetate	886	589	Sweet, fruity	AF+NBF	RI, aroma, S	4.12	3.65
2	Ethyl propionate	958	695	Fruity	AF+NBF	MS, RI, aroma, S	0.95	1.03
3	Ethyl 2-methylpropanoate	965	752	Nail polish	NBF	MS, RI, aroma, S	3.10	3.55
4	Isobutyl acetate	1014	768	Fruity	NBF	MS, RI, aroma, S	0.55	0.85
5	Ethyl butanoate	1037	817	Sweet, fruity	NBF	MS, RI, aroma, S	2.55	4.65
6	Ethyl 2-methylbutanoate	1050	841	Sweet, fruity	NBF	MS, RI, aroma, S	2.85	3.15
7	Ethyl 3-methylbutanoate	1062	856	Sweet, fruity	NBF	MS, RI, aroma, S	3.60	2.25
8	Isoamyl acetate	1134	878	Banana	NBF	MS, RI, aroma, S	4.20	4.35
9	Ethyl pentanoate	1142	903	Fruity	AF+NBF	MS, RI, aroma, S	2.25	4.32
10	Pentyl acetate	1184	926	Banana	NBF	MS, RI, aroma, S	0.22	0.35
11	3-Methylbutyl butanoate	1257	1061	Fruity	NBF	MS, RI, aroma, S	0.12	0.25
12	Ethyl hexanoate	1295	1025	Fruity	AF+NBF	MS, RI, aroma, S	1.90	3.65
13	Propyl hexanoate	1339	1081	Fruity	AF+NBF	MS, RI, aroma, S	0.20	0.20
14	Ethyl lactate	1373	825	Fruity	AF+NBF	MS, RI, aroma, S	0.65	0.42
15	Ethyl heptanoate	1394	1113	Fruity	NBF	MS, RI, aroma, S	0.45	0.72
16	Ethyl octanoate	1475	1200	Fruity	AF+NBF	MS, RI, aroma, S	3.95	3.52
17	Ethyl nonanoate	1595	1284	Fruity	NBF	MS, RI, aroma, S	1.20	0.65
18	Hexyl hexanoate	1621	1381	Fruity	NBF	MS, RI, aroma, S	0.20	0.22
19	Ethyl decanoate	1685	1391	Grape	NBF	MS, RI, aroma, S	2.62	2.25
20	Diethyl succinate	1711	1179	Sweet	AF+NBF	MS, RI, aroma, S	0.20	0.15
21	Ethyl benzoate	1722	1165	Fruity	NBF	MS, RI, aroma, S	1.35	1.20
22	Ethyl phenylacetate	1837	1254	Fruity	NBF	MS, RI, aroma, S	2.85	2.00
23	2-Phenethyl acetate	1871	1271	Rose	NBF	MS, RI, aroma, S	2.42	2.55
24	Ethyl 3-phenylpropanoate	1877	1345	Floral	NBF	MS, RI, aroma, S	1.20	4.42
25	Ethyl dodecanoate	1926	1581	Fruity	NBF	MS, RI, aroma, S	2.85	2.60
26	Ethyl tetradecanoate	2133	1790	Coconut	NBF	MS, RI, aroma, S	0.85	0.75
	**Alcohols**							
27	2-Butanol	1025	-	Wine	NBF	MS, RI, aroma, S	1.65	2.15
28	1-Propanol	1041	-	Alcoholic	AF+NBF	MS, RI, aroma, S	2.35	2.65
29	2-Methylpropanol	1095	620	Solvent	AF+NBF	MS, RI, aroma, S	3.05	3.52
30	1-Butanol	1149	669	Fruity	AF+NBF	MS, RI, aroma, S	2.02	2.22
31	1-Pentanol	1212	761	Fruity	NBF	MS, RI, aroma, S	0.65	0.75
32	3-Methylbutanol	1212	790	Whisky, burnt	AF+NBF	MS, RI, aroma, S	3.65	2.55
33	2-Heptanol	1324	901	Mushroom	NBF	MS, RI, aroma, S	1.00	0.85
34	1-Hexanol	1347	892	Fruity	NBF	MS, RI, aroma, S	0.85	0.95
35	3-Octanol	1437	992	Mushroom	NBF	MS, RI, aroma, S	0.25	0.25
36	1-Octen-3-ol	1456	964	Mushroom	NBF	MS, RI, aroma, S	3.65	2.20
37	1-Heptanol	1462	970	Green	NBF	MS, RI, aroma, S	0.20	0.10
38	1-Octanol	1559	1089	Fruity	NBF	MS, RI, aroma, S	1.02	0.85
39	1-Nonanol	1688	1168	Grass	NBF	MS, RI, aroma, S	1.15	1.05
40	Benzyl alcohol	1880	1033	Rose	NBF	MS, RI, aroma, S	0.75	0.82
41	β-Phenethyl alcohol	1959	1130	Rose, honey	AF+NBF	MS, RI, aroma, S	3.65	3.12
	**Aldehydes**							
42	2-Methyl propanal	816	550	Malty	NBF	RI, aroma, S	3.22	3.12
43	1,1-Dimethoxyethane	897	730	Fruity	NBF	MS, RI, aroma, S	4.02	3.65
44	3-Methylbutanal	922	621	Malty	NBF	MS, RI, aroma, S	3.03	1.95
45	Hexanal	1074	803	Grassy, green	NBF	MS, RI, aroma, S	3.25	3.02
46	1,1,3-Triethoxypropane	1303	1128	Fruity	AF+NBF	MS, RI, aroma, S	2.55	2.95
47	Decanal	1508	1228	Orange	NBF	MS, RI, aroma, S	0.35	0.32
48	Benzaldehyde	1589	975	Almond	NBF	MS, RI, aroma, S	1.12	0.95
49	Benzeneacetaldehyde	1698	1044	Honey	NBF	MS, RI, aroma, S	3.65	3.30
	**Ketones**							
50	2-Pentanone	1030	-	Fruity	NBF	MS, RI, aroma, S	1.23	0.22
51	2-Octanone	1296	997	Soap	NBF	MS, RI, aroma, S	3.22	3.20
52	Acetophenone	1660	1076	Floral	NBF	MS, RI, aroma, S	0.90	0.65
53	2-Pentadecanone	2072	-	Fruity	NBF	MS, RI, aroma, S	1.75	1.02
	**Acids**							
54	Acetic acid	1462	606	Vinegar	AF	MS, RI, aroma, S	4.12	4.05
55	Propanoic acid	1562	-	Rancid	AF	MS, RI, aroma, S	2.15	2.70
56	2-Methylpropanoic acid	1586	790	Sweaty	AF	MS, RI, aroma, S	3.25	3.22
57	Butanoic acid	1671	800	Sweaty	AF	MS, RI, aroma, S	3.55	3.95
58	3-Methylbutanoic acid	1707	835	Sweaty	AF	MS, RI, aroma, S	2.25	2.15
59	Pentanoic acid	1732	-	Sweat	AF	MS, RI, aroma, S	2.00	1.95
60	4-Methylpentanoic acid	1796	-	Rancid	AF	MS, RI, aroma, S	1.25	1.56
61	Hexanoic acid	1929	971	Sweaty	AF	MS, RI, aroma, S	2.32	1.75
62	Octanoic acid	2159	1280	Sweaty	AF	MS, RI, aroma, S	1.25	1.02
	**Phenols**							
63	Guaiacol	1874	1090	Smoky	NBF	MS, RI, aroma, S	3.25	1.22
64	4-Methylguaiacol	2016	1199	Smoky	AF+NBF	MS, RI, aroma, S	1.02	3.02
65	4-Ethylguaiacol	2091	1297	Spice	AF+NBF	MS, RI, aroma, S	0.95	2.15
66	4-Methyl phenol	2091	-	Medicinal	AF+NBF	MS, RI, aroma, S	1.00	0.95
67	4-Vinylguaiacol	2156	1311	Smoky	NBF	MS, RI, aroma, S	2.00	2.25
68	4-Ethyl phenol	2225	1172	Smoky	NBF	MS, RI, aroma, S	1.95	2.30
	**Terpenoids**							
69	Linalool	1542	1099	Floral	NBF	MS, RI, aroma, S	3.52	3.85
70	β-Damascenone	1759	1378	Rose	NBF	MS, RI, aroma, S	4.12	3.60
71	Geraniol	1858	1277	Rose	NBF	MS, RI, aroma, S	0.15	0.15
72	Geranylacetone	1864	1460	Sweet	NBF	MS, RI, aroma, S	0.20	0.15
73	β-Ionone	1917	1477	Floral	NBF	MS, RI, aroma, S	3.55	2.35
	**Others**							
74	2-Pentylfuran	1286	-	Sweet, fruity	NBF	MS, RI, aroma, S	2.05	1.12
75	Furfural	1471	845	Bread	NBF	MS, RI, aroma, S	1.52	1.05
76	2-Furan methanol	1666	813	Floral	NBF	MS, RI, aroma, S	0.25	0.20
77	γ-Nonanolactone	2012	1358	Coconut	NBF	MS, RI, aroma, S	2.65	2.05
78	Dimethyl trisulfide	1381	976	Cabbage	NBF	MS, RI, aroma, S	4.12	3.50
79	2-Thiophenecarboxaldehyde	1710	-	Almond	NBF	MS, RI, aroma, S	2.80	2.65

^a^ Retention indices determined by GC-MS on two different stationary phases (DB-FFAP and DB-5). ^b^ Odor quality perceived at the sniffing port. ^c^ The odorants were detected in fraction AF, acidic/water-soluble fraction; NBF, neutral/basic fraction.^d^ Identification based on RI (retention index), MS (mass spectrometry), aroma (odor description by comparison to the reference standards by GC-O), and S (standards).

**Table 2:** Quantitative methodological parameters of aroma compounds in Chinese *xiaoqu* Baijiu.

No.	Compound	Quantitative Ion (m/z)	IS ^a^	Slope	Intercept	R^2^	Recovery (%)	LOQ /μg/L
1	Ethyl acetate	61	IS1	0.0035	−0.0531	0.9995	93.46	3039.61
2	Ethyl propionate	57	IS1	0.0880	−3.5225	0.9973	91.32	721.15
3	Ethyl 2-methylpropanoate	71	IS1	0.3080	−0.0356	0.9993	87.79	47.95
4	Isobutyl acetate	43	IS1	0.3226	0.7735	0.9924	98.85	78.19
5	Ethyl butanoate	71	IS1	0.1670	0.1325	0.9985	85.07	125.58
6	Ethyl 2-methylbutanoate	57	IS1	0.3005	0.0479	0.9999	95.76	74.54
7	Ethyl 3-methylbutanoate	88	IS1	0.4875	0.0071	0.9995	85.05	5.68
8	Isoamyl acetate	70	IS1	0.0775	0.3756	0.9985	109.34	80.05
9	Ethyl pentanoate	88	IS1	0.3010	−0.1164	0.9992	82.74	26.68
10	Pentyl acetate	70	IS1	0.8020	−0.0135	0.9953	82.54	2.13
11	3-Methylbutyl butanoate	71	IS1	1.2970	0.0065	0.9963	105.76	0.38
12	Ethyl hexanoate	88	IS1	2.2610	1.5085	0.996	101.03	59.81
13	Propyl hexanoate	99	IS1	3.6525	−0.1232	0.9934	94.93	2.10
14	Ethyl lactate	45	IS1	0.6550	0.0325	0.9945	90.94	1.17
15	Ethyl heptanoate	88	IS1	4.5020	−0.0636	0.9933	93.04	9.80
16	Ethyl octanoate	88	IS1	1.1625	7.0776	0.9900	99.18	195.36
17	Ethyl nonanoate	88	IS1	5.5880	0.086	0.9965	88.20	3.41
18	Hexyl hexanoate	117	IS1	7.8150	−0.0281	0.9927	88.08	0.60
19	Ethyl decanoate	88	IS1	11.147	0.1139	0.9997	98.65	236.84
20	Diethyl succinate	101	IS1	0.2750	−0.8877	0.9975	105.78	73.66
21	Ethyl benzoate	105	IS1	5.7595	0.1188	0.9964	96.98	0.41
22	Ethyl phenylacetate	91	IS1	1.6570	−0.2062	0.9967	112.18	39.07
23	2-Phenethyl acetate	104	IS1	0.3597	0.0593	0.9996	96.49	7.89
24	Ethyl 3-phenylpropanoate	104	IS1	2.2125	0.1824	0.9987	103.68	7.81
25	Ethyl dodecanoate	88	IS1	6.5970	−0.2234	0.9978	83.70	4.05
26	Ethyl tetradecanoate	88	IS1	3.8505	−0.2599	0.9963	89.39	3.91
27	2-Butanol	45	IS2	0.0879	0.0581	0.9951	87.31	37.50
28	1-Propanol	59	IS2	0.0044	−0.1675	0.9995	84.21	7556.48
29	2-Methylpropanol	74	IS2	0.0043	−0.0098	0.9982	85.75	549.14
30	1-Butanol	56	IS2	0.0781	−0.4634	0.9952	105.22	363.80
31	1-Pentanol	55	IS2	0.2384	−0.0499	0.9955	103.65	15.62
32	3-Methylbutanol	55	IS2	0.0389	3.3248	0.9989	101.61	2320.31
33	2-Heptanol	55	IS2	0.8462	0.0115	0.9981	114.83	6.13
34	1-Hexanol	56	IS2	0.4043	1.1356	0.9978	111.05	132.38
35	3-Octanol	59	IS2	1.0170	0.0382	0.9987	113.69	9.62
36	1-Octen-3-ol	57	IS2	8.5931	0.0952	0.9991	107.11	1.40
37	1-Heptanol	70	IS2	2.8290	−0.0062	0.9965	114.48	0.43
38	1-Octanol	56	IS2	3.0323	0.6008	0.9973	86.28	5.67
39	1-Nonanol	56	IS2	10.2200	0.0967	0.9983	90.01	0.71
40	Benzyl alcohol	79	IS2	0.1822	0.0033	0.9956	83.53	3.78
41	β-Phenethyl alcohol	91	IS2	0.4217	0.3523	0.9972	85.43	58.36
42	2-Methyl propanal	72	IS4	0.0304	−0.0592	0.9973	91.71	69.64
43	1,1-Dimethoxyethane	73	IS4	0.0057	−0.1715	0.9978	83.29	3096.02
44	3-Methylbutanal	58	IS4	0.0364	−0.015	0.9974	85.70	210.62
45	Hexanal	56	IS4	0.2427	−0.0386	0.9973	104.62	8.10
46	1,1,3-Triethoxypropane	59	IS4	0.0234	−0.0125	0.9993	93.54	68.76
47	Decanal	57	IS4	3.5109	−0.0329	0.9974	8734.5	0.45
48	Benzaldehyde	106	IS4	0.9092	0.0855	0.9959	118.17	8.15
49	Benzeneacetaldehyde	91	IS4	0.2074	−0.0312	0.9997	87.34	19.95
50	2-Pentanone	86	IS4	0.0542	−0.0248	0.9996	87.77	27.50
51	2-Octanone	58	IS4	3.8946	−0.1028	0.9992	108.62	2.23
52	Acetophenone	105	IS4	2.0508	0.0256	0.9958	96.17	0.36
53	2-Pentadecanone	58	IS4	63.259	−0.0799	0.9963	81.80	0.20
54	Acetic acid	60	IS3	0.0082	−0.0565	0.9996	108.72	8260.31
55	Propanoic acid	74	IS3	0.0235	−0.0334	0.9956	104.88	2970.00
56	2-Methylpropanoic acid	73	IS3	0.0851	−0.0764	0.9963	88.10	742.16
57	Butanoic acid	60	IS3	0.1737	−0.0698	0.9989	108.42	523.46
58	3-Methylbutanoic acid	60	IS3	0.5851	−0.0283	0.999	107.91	105.76
59	Pentanoic acid	73	IS3	0.2789	−0.1994	0.9982	96.28	1166.94
60	4-Methylpentanoic acid	57	IS3	1.1236	−0.0169	0.9962	91.06	7.84
61	Hexanoic acid	60	IS3	2.1683	0.0657	0.9995	100.40	69.61
62	Octanoic acid	73	IS3	3.0605	−0.0883	0.9987	99.70	21.70
63	Guaiacol	109	IS5	0.6820	0.0016	0.9993	96.06	1.83
64	4-Methylguaiacol	138	IS5	1.1433	−0.0054	0.9979	106.42	0.67
65	4-Ethylguaiacol	137	IS5	3.1906	−0.0012	0.9989	102.74	0.28
66	4-Methyl phenol	107	IS5	0.8881	−0.0029	0.9977	107.47	1.48
67	4-Vinylguaiacol	150	IS5	0.3140	−0.0226	0.9969	99.26	4.00
68	4-Ethyl phenol	107	IS5	2.0922	−0.0002	0.9982	104.70	0.07
69	Linalool	71	IS4	2.3080	−0.0111	0.9999	98.18	1.57
70	β-Damascenone	69	IS4	0.0225	0.0001	0.9972	98.63	16.00
71	Geraniol	69	IS4	2.8196	0.0086	0.9962	92.21	0.08
72	Geranylacetone	69	IS4	12.5120	0.0091	0.9985	97.76	0.08
73	β-Ionone	177	IS4	5.8592	0.0075	0.9954	87.96	0.19
74	2-Pentylfuran	81	IS4	3.8924	−0.7053	0.9982	101.65	13.70
75	Furfural	96	IS4	0.0429	0.1024	0.9964	107.74	224.30
76	2-Furan methanol	98	IS4	0.0064	−0.0137	0.9980	83.96	120.40
77	γ-Nonanolactone	85	IS4	1.0395	−0.0052	0.9980	86.55	0.36
78	Dimethyl trisulfide	126	IS4	0.4135	0.0003	0.9994	92.86	0.25
79	2-Thiophenecarboxaldehyde	111	IS4	0.0125	0.0002	0.9994	87.59	9.71

^a^ IS, internal standard; IS1, 2-phenylethyl acetate-D_3_; IS2, n-hexyl-D_13_ alcohol; IS3, 2,2-dimethyl-propanoic acid; IS4, 2-octanol; IS5, 2-methoxy-D_3_-phenol.

**Table 3:** Concentrations of aroma compounds, odor thresholds, and odor activity values (OAVs) in Chinese *xiaoqu* Baijiu.

No.	Compound	Concentration (μg/L)		Threshold ^a^	OAV		Concentration Ratio
		M	T	(μg/L)	M	T	
16	Ethyl octanoate	6542.88 ± 194.20	3042.84 ± 107.10	12.9 ^b^	507.20	235.88	2.15 ^g^
44	3-Methylbutanal	5077.61 ± 201.98	3140.96 ± 99.06	16.5 ^b^	307.74	190.36	1.62 ^g^
70	β-Damascenone	21.48 ± 1.22	9.37 ± 0.23	0.12 ^c^	178.98	78.11	2.29 ^g^
78	Dimethyl trisulfide	70.04 ± 3.75	53.81 ± 1.59	0.41 ^b^	161.22	116.14	1.38 ^g^
43	1,1-Dimethoxyethane	293,200.02 ± 16,250.65	194,930.69 ± 12,890.61	2090 ^d^	140.03	90.33	1.50 ^g^
8	Isoamyl acetate	9106.74 ± 855.52	12,434.91 ± 713.97	94 ^b^	96.88	132.29	1.37 ^h^
12	Ethyl hexanoate	2683.21 ± 121.80	7395.99 ± 579.91	55.3 ^b^	48.52	133.74	2.76 ^h^
1	Ethyl acetate	1,189,626.20 ± 28,686.27	1,093,055.49 ± 74,210.60	32600 ^b^	36.49	33.53	1.09 ^g^
7	Ethyl 3-methylbutanoate	155.12 ± 15.12	45.24 ± 2.64	6.9 ^b^	22.48	6.56	3.43 ^g^
9	Ethyl pentanoate	545.39 ± 50.56	3075.6 ± 276.15	26.8 ^b^	20.35	114.76	5.64 ^h^
45	Hexanal	464.28 ± 23.31	350.84 ± 18.68	25.5 ^b^	18.21	13.76	1.32 ^g^
59	Pentanoic acid	6731.23 ± 536.16	7721.93 ± 122.13	389 ^b^	17.30	19.85	1.15 ^h^
36	1-Octen-3-ol	94.04 ± 8.29	34.87 ± 2.26	6.12 ^c^	15.37	5.70	2.70 ^g^
5	Ethyl butanoate	1158.52 ± 98.27	20,740.96 ± 684.53	81.5 ^b^	14.21	254.49	17.90 ^h^
28	1-Propanol	676,572.64 ± 62,948.37	858,160.14 ± 43,057.77	54,000 ^b^	12.53	15.89	1.27 ^h^
69	Linalool	162.25 ± 16.03	190.93 ± 5.00	13.1 ^e^	12.39	14.57	1.18 ^h^
49	Benzeneacetaldehyde	2445.78 ± 61.59	1404.18 ± 15.74	262 ^d^	9.34	5.36	1.74 ^g^
29	2-Methylpropanol	236,239.43 ± 18,833.05	327,289.50 ± 20,690.96	28,300 ^c^	8.35	11.57	1.39 ^h^
6	Ethyl 2-methylbutanoate	145.14 ± 8.87	149.22 ± 3.49	18 ^b^	8.06	8.29	1.03 ^h^
63	Guaiacol	102.93 ± 6.09	51.96 ± 2.19	13.4 ^b^	7.68	3.88	1.98 ^g^
57	Butanoic acid	6842.96 ± 187.62	10,424.39 ± 404.47	964 ^b^	7.10	10.82	1.52 ^h^
27	2-Butanol	339,921.21 ± 8741.68	665,096.28 ± 13,098.88	50,000 ^b^	6.81	13.29	1.95 ^h^
32	3-Methylbutanol	1,165,477.58 ± 90,842.60	757,555.98 ± 66,411.82	179,000 ^b^	6.51	4.23	1.54 ^g^
30	1-Butanol	17,586.32 ± 1058.58	27,280.65 ± 1578.84	2730 ^b^	6.44	9.99	1.55 ^h^
25	Ethyl dodecanoate	2507.65 ± 244.90	1471.96 ± 106.06	400 ^f^	6.27	3.68	1.70 ^g^
58	3-Methylbutanoic acid	6300.25 ± 75.10	5426.10 ± 351.74	1050 ^b^	6.00	5.17	1.16 ^g^
19	Ethyl decanoate	5867.76 ± 366.2	5123.68 ± 182.18	1120 ^b^	5.24	4.57	1.15 ^g^
3	Ethyl 2-methylpropanoate	240.10 ± 15.57	262.45 ± 15.12	57.5 ^b^	4.18	4.56	1.09 ^h^
73	β-Ionone	5.28 ± 0.20	3.86 ± 0.31	1.3 ^d^	4.06	2.97	1.37 ^g^
14	Ethyl lactate	510,767.23 ± 970.08	484,485.04 ± 387.49	128,000 ^b^	3.99	3.79	1.05 ^g^
60	4-Methylpentanoic acid	567.06 ± 16.66	835.88 ± 30.11	144 ^c^	3.94	5.80	1.47 ^h^
67	4-Vinylguaiacol	691.08 ± 24.02	804.60 ± 16.15	209 ^b^	3.30	3.84	1.16 ^h^
56	2-Methylpropanoic acid	5131.08 ± 428.69	4225.58 ± 286.11	1580 ^d^	3.25	2.68	1.21 ^g^
54	Acetic acid	557,164.75 ± 54,070.93	426,675.42 ± 22,473.95	200,000 ^b^	2.79	2.14	1.31 ^g^
68	4-Ethyl phenol	1379.60 ± 42.75	3573.64 ± 58.21	618 ^b^	2.24	5.78	2.59 ^h^
23	2-Phenethyl acetate	1870.82 ± 198.57	1970.58 ± 188.82	909 ^b^	2.06	2.17	1.05 ^h^
61	Hexanoic acid	5046.77 ± 331.75	3916.83 ± 236.21	2520 ^b^	2.00	1.55	1.29 ^g^
46	1,1,3-Triethoxypropane	7268.03 ± 576.60	12,143.02 ± 793.82	3700 ^b^	1.97	3.28	1.67 ^h^
77	γ-Nonanolactone	172.01 ± 6.48	49.76 ± 1.13	90.7 ^b^	1.90	0.55	3.46 ^g^
55	Propanoic acid	31,045.80 ± 2132.72	37,776.75 ± 1305.67	18,200 ^b^	1.71	2.08	1.22 ^h^
24	Ethyl 3-phenylpropanoate	199.54 ± 6.45	2470.88 ± 120.61	130 ^b^	1.54	19.01	12.34 ^h^
65	4-Ethylguaiacol	178.16 ± 5.15	387.86 ± 2.40	123 ^b^	1.44	3.16	2.18 ^h^
41	β-Phenethyl alcohol	40,101.93 ± 2462.57	23,215.7 ± 1252.70	28,900 ^b^	1.39	0.80	1.73 ^g^
42	2-Methyl propanal	1708.38 ± 140.81	1525.56 ± 149.01	1300 ^b^	1.31	1.14	1.12 ^g^
64	4-Methylguaiacol	290.22 ± 18.08	694.76 ± 36.65	315 ^b^	0.92	2.21	2.39 ^h^
62	Octanoic acid	2374.17 ± 36.35	1423.26 ± 90.68	2700 ^b^	0.88	0.53	1.67 ^g^
10	Pentyl acetate	142.56 ± 7.20	288.16 ± 7.20	180 ^d^	0.80	1.60	2.02 ^h^
2	Ethyl propionate	10,868.83 ± 544.43	12,827.82 ± 1141.29	19,000 ^b^	0.57	0.68	1.18 ^h^
47	Decanal	40.61 ± 3.19	60.12 ± 4.03	70.8 ^d^	0.57	0.85	1.48 ^h^
34	1-Hexanol	2740.72 ± 90.84	3820.35 ± 140.62	5370 ^d^	0.50	0.70	1.39 ^h^
33	2-Heptanol	685.65 ± 16.11	85.65 ± 5.60	1430 ^d^	0.48	0.06	8.01 ^g^
66	4-Methyl phenol	55.82 ± 3.26	39.10 ± 6.93	167 ^b^	0.34	0.24	1.43 ^g^
51	2-Octanone	64.45 ± 5.99	58.59 ± 5.80	250 ^c^	0.26	0.23	1.10 ^g^
39	1-Nonanol	187.91 ± 11.48	136.68 ± 8.94	806 ^d^	0.23	0.17	1.37 ^g^
35	3-Octanol	86.50 ± 5.15	106.75 ± 7.84	393 ^c^	0.22	0.27	1.23 ^g^
52	Acetophenone	50.55 ± 2.91	6.82 ± 0.41	256 ^d^	0.20	0.03	7.41 ^g^
75	Furfural	6568.22 ± 651.46	2581.56 ± 113.18	44,000 ^b^	0.15	0.06	2.54 ^g^
22	Ethyl phenylacetate	44.49 ± 2.99	35.24 ± 2.59	407 ^b^	0.11	0.09	1.26 ^g^
17	Ethyl nonanoate	326.85 ± 32.41	173.76 ± 15.58	3200 ^b^	0.10	0.05	1.88 ^g^
72	Geranylacetone	23.83 ± 1.82	18.81 ± 0.29	267 ^f^	0.09	0.07	1.27 ^g^
48	Benzaldehyde	317.00 ± 23.26	151.23 ± 1.24	4200 ^b^	0.08	0.04	2.10 ^g^
76	2-Furan methanol	4178.60 ± 104.80	2012.85 ± 46.29	54,700 ^c^	0.06	0.04	1.56 ^g^
20	Diethyl succinate	18,214.34 ± 1109.16	11,493.28 ± 865.21	353,000 ^b^	0.05	0.03	1.58 ^g^
71	Geraniol	5.78 ± 0.19	3.01 ± 0.29	120 ^f^	0.05	0.03	1.92 ^g^
11	3-Methylbutyl butanoate	35.15 ± 3.45	431.55 ± 38.24	915 ^d^	0.04	0.47	12.28 ^h^
38	1-Octanol	33.00 ± 1.37	13.56 ± 0.86	1100 ^c^	0.03	0.01	2.43 ^g^
31	1-Pentanol	1052.35 ± 103.83	1639.97 ± 135.46	37,400 ^b^	0.03	0.04	1.56 ^h^
40	Benzyl alcohol	1024.33 ± 40.75	2302.44 ± 81.79	40,900 ^b^	0.03	0.06	2.25 ^h^
21	Ethyl benzoate	22.15 ± 2.02	3.78 ± 0.23	1400 ^b^	0.02	<0.01	5.86 ^g^
4	Isobutyl acetate	16.38 ± 1.39	49.14 ± 4.58	922 ^b^	0.02	0.05	3.00 ^h^
15	Ethyl heptanoate	195.83 ± 18.23	204.63 ± 13.15	13,200 ^b^	0.01	0.02	1.04 ^h^
37	1-Heptanol	174.40 ± 2.79	106.98 ± 6.02	26,600 ^d^	0.01	<0.01	1.63 ^g^
13	Propyl hexanoate	34.25 ± 2.54	129.31 ± 8.21	13,000 ^d^	<0.01	0.01	3.78 ^h^
18	Hexyl hexanoate	3.96 ± 0.25	15.73 ± 1.16	1890 ^d^	<0.01	0.01	3.97 ^h^
26	Ethyl tetradecanoate	835.26 ± 44.31	454.07 ± 22.18	494,000 ^b^	<0.01	<0.01	1.84 ^g^
50	2-Pentanone	775.20 ± 60.42	346.25 ± 10.92	-			2.24 ^g^
74	2-Pentylfuran	751.32 ± 57.57	466.86 ± 29.76	-			1.61 ^g^
79	2-Thiophenecarboxaldehyde	858.04 ± 47.96	556.41 ± 16.91	-			1.54 ^g^
53	2-Pentadecanone	3.84 ± 0.27	3.40 ± 0.09	-			1.13 ^g^

^a^ Odor thresholds were taken from reference. ^b^ Odor thresholds taken from reference [22,23]. ^c^ Odor thresholds taken from reference [24]. ^d^ Odor thresholds taken from reference [25]. ^e^ Odor thresholds taken from reference [31]. ^f^ Odor thresholds taken from reference [13]. ^g^ The concentration ratio of M to T. ^h^ The concentration ratio of T to M.Identified odorants with high OAVs can also be used as indicators to assess aroma differences objectively in the two samples. Among the compounds with OAVs ≥ 1, we found that the concentrations of ethyl octanoate, *β*-damascenone, 3-methylbutanal, ethyl 3-methylbutanoate, benzeneacetaldehyde, 1-octen-3-ol, guaiacol, 1,1-dimethoxyethane, 3-methylbutanol, ethyl dodecanoate, *γ*-nonanolactone, and *β*-phenethyl alcohol, were 1.50–17.9 times higher in the M than in the T sample. In contrast, the concentrations of ethyl butanoate, ethyl pentanoate, ethyl hexanoate, 1-butanol, 2-butanol, butanoic acid, 1,1,3-triethoxypropane, 4-ethylphenol, 4-methylguaiacol, ethyl 3-phenylpropanoate, and 4-ethylguaiacol were 1.52–24.77 times higher in the T sample than in the M sample. The 23 aroma compounds might be potential compounds responsible for the differences in the aroma profile between M and T samples. Figure 2 shows the concentration of the 12 aroma-active compounds (concentration ratio ≥ 2) in the M and T samples.

## Data Availability

The data that support the findings of this study are available from the corresponding author upon request.
